# Scientific validation of the antimicrobial and antiproliferative potential of *Berberis aristata* DC root bark, its phytoconstituents and their biosafety

**DOI:** 10.1186/s13568-019-0868-4

**Published:** 2019-09-11

**Authors:** Henna Sood, Yashwant Kumar, Vipan Kumar Gupta, Daljit Singh Arora

**Affiliations:** 1 Microbial Technology Laboratory, Department of Microbiology, Guru Nanak Dev University, Amritsar, 143005 India; 2 National Salmonella & Escherichia Centre and Diagnostic Reagents Laboratory, Central Research Institute, Kasauli, H.P. 173204 India; 3 Department of Veterinary Pathology, Dr. G.C.Negi College of Veterinary and Animal Sciences, CSK Himachal Pradesh Krishi Vishvavidyalaya, Palampur, H.P. 176062 India

**Keywords:** Antimicrobial, Acute oral toxicity, *Berberis aristata*, Biosafety, Phytoconstituents, Post-antibiotic effect

## Abstract

*Berberis aristata* is an important part of traditional healing system from more than 2500 years. The aqueous extract of *Berberis aristata* root bark displayed broad spectrum activity against 13 test pathogens, ranging from 12 to 25 mm. In classical optimization, 15% concentration prepared at 40 °C for 40 min was optimal and thermostable. Statistical optimization enhanced the activity by 1.13–1.30-folds. Ethyl acetate was the best organic solvent to elute out the potential compound responsible for antimicrobial activity. Diterpenes were the most abundant phytoconstituent (15.3%) and showed broad spectrum antimicrobial activity ranging from 16.66 to 42.66 mm. Ethyl acetate extract displayed the lowest minimum inhibitory concentration (0.05–1 mg/mL), followed by diterpenes (0.05–5 mg/mL) and flavonoids (0.05–10 mg/mL). The test extracts were microbicidal in nature and showed a prolonged post antibiotic effect ranging from 2 to 8 h. They were found to be biosafe as per Ames and MTT assay. The in vitro cytotoxicity evaluation of diterpenes against L20B, RD and Hep 2 cell lines revealed its IC_50_ ranging from 245 to 473 µg/mL. Acute oral toxicity of diterpenes on Swiss albino mice did not show any changes in behavioral pattern, body weight, biochemical parameters as well as organs’ architecture. The study thus indicates *B. aristata* could be a potential candidate for development of potent drug owing to its antimicrobial potential and biosafe profile.

## Introduction

Over the past few years the emergence of highly infectious diseases and resistant microbial strains are adding up to the woes of human health (Li and Webster 2018). Thus, the alarming challenge for physicians and pharmacists is the need to develop alternative approaches and search for new antimicrobials from natural sources, which include microbial sources such as bacteria (Kos et al. 2011) and fungi (Sondergaard et al. 2016) as well as non-microbial sources such as animals (Burrowes et al. 2004) and plants (Sibanda and Okoh 2007; Ayukekbong et al. 2017). Among these, plants have provided a ray of hope for obtaining novel antimicrobial compounds (Zhao et al. 2013; Farjana et al. 2014; Ruma et al. 2015; Arora and Sood 2017; Zheljazkov et al. 2017; Oluwasina et al. 2019).

The genus *Berberis* (Family: *Berberidaceae*) has occupied an important position in various traditional medicinal systems. It comprises of approximately 500 species of pharmacological importance, distributed within various geographical regions of Asia, Europe and America. Different species such as *B. vulgaris, B. darwinii, B. lyceum, B. orthobotrys, B. chitria, B. aristata, B. heterophylla, B. integerrima, B. aquifolium, B. lyceum*, *B. aetnensii, B. umbellate* etc. have been used by various tribes in different traditional medical systems worldwide. Its different species possess stomachic, astringent, antiperiodic, diaphoretic properties. It is also useful in the treatment of jaundice, dysentery, leishmaniasis, malaria, gall stones, enlargement of spleen and cardiovascular disorders etc. Various extracts and purified compounds from almost all parts of these plants have been reported to possess biological activities including anti-convulsion, hypoglycemic, anticancer, anti-histaminic, antifatigue, anticoagulant, immuno-stimulant, antidiabetic, osteolytic, antipyretic, antimicrobial, antioxidant, anti-inflammatory, hypotensive, anti-cholestrolemic, CNS-depressant, anti-nociceptive, anti-cholinergic activities etc. (Srivastava et al. 2015; Abushouk et al. 2017). These pharmacological properties make them an important part of the polyherbal formulations used for the treatment of several diseases and disorders (Mokhber-Dezfuli et al. 2014).

*Berberis aristata* is one such important species. It is a spinous herb, also known as Indian barberry or tree turmeric (English), darhaldi, dar-hald, rasaut (Hindi) and daruharidra (Sanskrit), and is native to the north Himalayan region (Mazumder et al. 2011). It is used for a variety of purposes like fuel wood, animal fodder, printing and dyeing industry. Various parts of the plant (root, stem, bark and fruits) have been traditionally used in different health issues such as wound healing, inflammation, ENT infections, skin disease, jaundice, vaginal disorders, diarrhea and infection of eyes (Rashmi et al. 2008). It is also known for its antimicrobial (Sharma et al. 2011; Saravanakumar et al. 2014; Saxena et al. 2014), anti-inflammatory (Gupta et al. 2008), hepatoprotective (Unkeshwar et al. 2013), antidiabetic (Ahmad et al. 2012), antioxidant (Das et al. 2015) and anticarcinogenic (Pai et al. 2012) properties to name a few.

Though the traditional medicinal plants have long been used by the ancestors for the treatment of many diseases, but still little is known about their toxicity and biosafety (Jothy et al. 2011). Toxicity evaluation of medicinal plants is of vital importance, when considering public health protection, as it helps identify the range of doses and also reveal the possible clinical signs elicited by the compound under investigation. It is a useful parameter to investigate the therapeutic index of compounds which have got the potential to be developed as drugs. Upon extensive search of literature, it has been noted that *Berberis aristata* and its related species have been less explored in terms of their antimicrobial potential, isolation of antimicrobial compounds as well as the biosafety profile (Anubhuti et al. 2011; Sharma et al. 2011; Daud et al. 2013; Irshad et al. 2013; Saravanakumar et al. 2014; Saxena et al. 2014; Malik et al. 2017; Rizwan et al. 2017).

Keeping this in mind, the present study has been carried out to standardize the various physiochemical parameters such as extract concentration, extraction temperature, extraction time, extract pH and various filtration strategies for obtaining the best expression of antimicrobial activity of *Berberis aristata* DC root bark by first varying one parameter at a time (classical method) and then the statistical optimization of these parameters. The best organic solvent and the major group of phytoconstituents were established and along with standard antibiotics, were tested for their minimal inhibitory concentration (MIC), viable cell count (VCC) and post antibiotic effect (PAE). In addition, diterpenes‚ which have shown the highest inhibitory potential among tested phytoconstituents‚ were further tested for their cytotoxicity in vitro on three different tumor cell lines including RD, L20B and Hep 2. The test extracts were also subjected to in vitro biosafety evaluation by Ames test and MTT assay. Apart from in vitro biosafety assays, toxicity results from animals are crucial in definitively judging the safety of medicinal plants. Therefore, to ensure public health protection and rule out any possibilities of adverse effects on human health, the in vivo safety of the diterpenes was determined by acute oral toxicity test in Swiss albino mice so as to confirm its consumability when developed in the form of a drug.

## Materials and methods

### Plant material and its extract preparation

*Berberis aristata* root bark was obtained locally from Amritsar, Punjab. The formal identification of the plant material was carried out by Dr. Amarjit Singh Soodan (Associate Professor), Department of Botanical and Environmental Sciences, Guru Nanak Dev University (Amritsar), Punjab and was thereby deposited in the above said department wide accession no. 766-768 Bot. & Env. Sc. dated 02/07/15. The plant name has been checked with http://www.theplantlist.org. The plant material was surface sterilized, air dried and powdered. An aqueous extract was then prepared by aseptically suspending a known amount of powdered plant material into a known volume of sterile distilled water and extracting it in a hot water bath at 40 °C for 20 min. The extracted material was then vacuum filtered using whatman filter paper No.1 and subjected to antimicrobial testing by agar well diffusion assay (ADA) (Arora and Sood 2017). The chemicals and antibiotics were procured from Hi-Media Pvt. Ltd., India.

### Microorganisms used in the study

The reference strains of bacteria and yeast such as *Enterococcus faecalis* (MTCC 439), *Staphylococcus aureus* (MTCC 740), *Staphylococcus epidermidis* (MTCC 435), *Escherichia coli* (MTCC 119), *Klebsiella pneumoniae* 1 (MTCC 109), *Klebsiella pneumoniae* 2 (MTCC 530), *Pseudomonas aeruginosa* (MTCC 741), *Salmonella typhimurium* 1 (MTCC 98), *Salmonella typhimurium* 2 (MTCC 1251), *Shigella flexneri* (MTCC 1457) and yeast strains such as *Candida albicans* (MTCC 227) and *Candida tropicalis* (MTCC 230) were procured from microbial type culture collection (MTCC), Institute of Microbial Technology (IMTECH), Chandigarh (Arora and Sood 2017). Besides these standard strains, a clinical isolate of MRSA was procured from Post Graduate Institute of Medical Education and Research, Chandigarh (India). All the cultures were preserved in the glycerol stock at − 80 °C.

### Inoculum preparation and antimicrobial screening

The inoculum was prepared as per McFarland standard (Arora and Sood 2017). The 10% plant aqueous extract was initially screened for the presence of antimicrobial activity against the reference strains by agar well diffusion assay (Arora and Onsare 2014c). Three antibiotics, i.e., two antibacterial antibiotics (gentamicin, chloramphenicol) with different spectrum of inhibitory activity and one antifungal antibiotic (amphotericin B) were used to compare their antimicrobial activity, in terms of zone of inhibition, with that of the aqueous extract and phytoconstituents extracted from *Berberis aristata*.

### Optimization of physiochemical parameters using one-factor-at-a-time approach

Parameters such as extract concentration, extraction temperature, extraction time, pH and filtration methods were optimized using classical approach as described previously in Arora and Sood (2017).

### Statistical optimization of the parameters by response surface methodology (RSM) using Box–Behnken design

On the basis of results obtained from the one- factor-at- a-time method, various parameters such as extraction temperature, extraction time and extract concentration were taken as the independent variables so as to find out the effect of the variables’ interaction on the antimicrobial potential. Each variable was studied at three different levels (− 1, 0, + 1), which were 40 °C, 50 °C and 60 °C (extraction temperature); 20 min, 40 min and 60 min (extraction time), and 10%, 15% and 20% (concentration). The experimental design included 17 tubes with five replicates having all the variables at their central coded values, where the zone of inhibition (in mm) for two Gram positive (MRSA, *S. aureus*) and two Gram negative (*E. coli, K. pneumoniae* 1) bacteria was taken as the response G_(1–4)_. The mathematical relationship of response G (for each parameter) and independent variable X (X_1_: concentration; X_2_: time; X_3_: temperature) was calculated by the following quadratic model equation:

$$G_{{\left( { 1{-} 4} \right)}} = \beta_{0} + \beta_{ 1} X_{ 1} + \beta_{ 2} X_{ 2} + \beta_{ 3} X_{ 3} + \beta_{ 1 1} X_{ 1}^{ 2} + \beta_{ 2 2} X_{ 2}^{ 2} + \beta_{ 3 3} X_{ 3}^{ 2} + \beta_{ 1 2} X_{ 1} X_{ 2} + \beta_{ 1 3} X_{ 1} X_{ 3} + \beta_{ 2 3} X_{ 2} X_{ 3} ,$$

where *G* is the predicted response; *β*_0_ intercept; *β*_1_, *β*_2_, and *β*_3_ linear coefficients; *β*_11_, *β*_22_, and *β*_33_ squared coefficient and *β*_12_, *β*_13_, and *β*_23_ interaction coefficients. The Analysis of the experimental data was done from the response surface graphs using MINITAB software version 11 (Box and Behnken 1960; Kaur et al. 2015).

### Thermostability studies

Fifteen percent aqueous extract of the plant was exposed to a temperature range of 60–100 °C for 1 h and any loss in activity was worked out by comparing it with that of untreated plant extract.

### Determination of the best organic solvent

The 15% aqueous extract (100 mL) was extracted thrice with an equal volume (100 mL) of the organic solvents, i.e., chloroform, hexane, ethyl acetate and butanol (s d fine chem-limited). After every extraction cycle, the organic layers were collected, pooled and then concentrated at 45 °C using a rotary evaporator (Buchi Rotavapor R-210). The concentrates for each solvent were finally reconstituted in 30% DMSO (s d fine chem-limited) to give the respective stock solutions of 40.15 mg/mL, 38.82 mg/mL, 42.33 mg/mL and 44.03 mg/mL. Their antimicrobial potency was thus ascertained by the agar well diffusion assay (Arora and Sood 2017).

### Qualitative and quantitative profiling of phytoconstituents

The powdered plant material was qualitatively analyzed for the presence/absence of the major groups of phytoconstituents by suspending it in a particular solvent as per the protocol used for each particular phytoconstituent (protocols mentioned in Additional file 1). They were then isolated using standard quantitative techniques, dissolved in a known volume of 30% DMSO and then tested for their antimicrobial activity (Kaur and Arora 2009; Ezeonu and Ejikeme 2016; Arora and Sood 2017). The qualitative and quantitative estimation of the phytoconstituents was carried out using standard protocols as mentioned in Additional file 1. The results obtained were then compared to the standard antibiotics like gentamicin, chloramphenicol (for bacteria) and amphotericin B (for yeast).

### Minimum inhibitory concentration (MIC)

The MIC of all the test extracts was assessed by the agar dilution method (Arora and Sood 2017). The various concentrations, ranging from 0.5 to 25 mg/mL (aqueous extract), 0.01–5 mg/mL (organic extract) and 0.01–10 mg/mL (phytoconstituents), were prepared to determine their MIC values. The MICs were compared to that of standard antibiotics (gentamicin, chloramphenicol and amphotericin B). The diluent (30% DMSO) without the test compound was considered as the negative control.

### Viable cell count (VCC) studies

To work out the kill time, the stock solutions (as described previously) of different test extracts were prepared and the assay was performed as per protocol followed in Sood et al. (2015).

### Determination of the post antibiotic effect of the ethyl acetate extract, flavonoids and diterpenes

The PAE of the ethyl acetate extract, flavonoids and diterpenes was performed according to Babakhani et al. (2011) and Raja et al. (2011). The test compounds were mixed in equal ratio with the suspension of respective test organism and incubated at 37 °C for 2 h, after which the drug activity was halted by diluting the mixture. From this diluted suspension, samples (0.1 mL) were taken and plated at an interval of 2 h up to 24 h so as to obtain the CFU count. The culture without the test compound was taken as positive. The values was worked out as PAE = T–C, as T (denotes test) is the time required for the CFU count to increase by 1 log_10_CFU/mL whereas C represents the control.

### Biosafety evaluation of *Berberis aristata* root bark by Ames test and MTT assay

The test extracts were subjected to Ames mutagenicity test (by plate incorporation method) and MTT toxicity assay as described earlier in Arora and Sood (2017). In Ames Mutagenicity test, the aqueous extract, ethyl acetate extract and the phytoconstituents (at their MIC concentrations) were mixed with an equal volume of the diluted (10^−3^) culture of *Salmonella typhimurium* (MTCC 1251, IMTECH, Chandigarh) in a top agar medium containing 0.5 mM histidine–biotin mixture (1:1 ratio). Here, Sodium azide (5 µL of 17.2 mg/mL) was used as a positive control.

In MTT toxicity assay, 100 µL of the diluted blood cell suspension was added in each well and incubated at 37 °C for overnight, followed by addition of 200 µL of the extract (aqueous extract, ethyl acetate extract and the phytoconstituents) and incubation for 24 h. Then 20 µL MTT solution (5 mg/mL) was added to each well and incubated further for 3.5 h at 37 °C on orbital shaker at 60 rpm. After incubation, the supernatant was removed carefully and 50 µL DMSO was added to each well to dissolve the formazan crystals. The absorbance was measured at 590 nm using an automated microplate reader (Biorad 680-XR, Japan). The wells with untreated cells served as control.

### In vitro cytotoxicity testing using RD, L20B and Hep2 cell lines by MTT assay

The cytotoxic effect of the most active phytoconstituent (diterpenes) of *Berberis aristata* root bark was studied against three cell lines [RD (Human Rhabdomyosarcoma), L20B (Diploid mouse lung cell line) and Hep 2 (Human larynx epidermoid carcinoma)] by MTT assay as described earlier in (Al-Asady et al. 2014; Das and Devi 2015) with slight modifications, as per protocol given in Additional file 2. The cell lines were procured from Central Research Institute (C.R.I), Kasauli, Himachal Pradesh, India. Twofold serial dilutions ranging from 10 to 0.039 mg/mL were used in the experimentation. The IC_50_ values were calculated from the dose–response curve generated for each cell line.

### Acute oral toxicity study of *Berberis aristata* diterpenes in Swiss albino mice

In order to validate the non-toxicity of the diterpenes in animal models, acute oral toxicity was studied as described previously (Jothy et al. 2011; Ping et al. 2013) with slight modifications. The experiment was conducted at Central Research Institute, Kasauli, Himachal Pradesh, India. For the study, healthy Swiss albino mice (males and females), weighing between 25 and 35 g and aged 8 to 10 weeks, were obtained from the animal house, Central Research Institute, Kasauli (H.P.). The mice were divided into 4 groups: 6 males in the test male group; 6 females in the test female group, 6 males in the control male group and 6 females in the control female group. The treatment or test group was given a single dose (5000 mg/kg) of the diterpenes (dissolved in normal saline) and the control group was given normal saline lacking the diterpenes. Prior to dosage by oral route, the mice were fasted overnight but were allowed free access to water. Following the fasting period, body weight of the mice was determined and a single dose was calculated in reference to the body weight, as the volume of the compound (diterpenes) to be given to the mice is 10 mL/kg. The mice were kept under continuous observation for any signs of toxicity and mortality, firstly at 4 h and 24 h interval, and then daily for a period of 14 days. During this period the surviving animals were weighed and visually observed for changes in behavioral pattern, physical appearance, injury and any signs of illness. On the 15th day, the final weights of mice were noted and they were subsequently anaesthetized using xylaxine and ketamine (5 mg/kg and 2.5 mg/kg b.wt. respectively). For biochemical analysis, the blood samples were collected (from both treated and control group) via cardiac puncture in non-heparinized tubes. Subsequently, the serum was separated and analyzed for alanine aminotransferase (ALT), alkaline phosphatase (ALP), aspartate aminotransferase (AST), total bilirubin (TBIL), urea and creatinine levels. Following the blood collection, all the animals were sacrificed by an overdose of anesthesia. The vital organs (mainly liver, kidney and heart) were removed, cleaned with saline and macroscopically examined for any lesions, followed by their histopathological examination. The experimental procedure was approved by the Committee for the Purpose of Control and Supervision of Experiments on Animals (CPCSEA) (No. CPCSEA/IAEC/CRI/14-114-2016), which was performed according to the Organization of Economic Co-operation and Development (OECD) guideline 420 used for the testing of chemicals (see Additional file 3).

### Data analysis

The experiments were done in duplicate sets and repeated three times. The values in most experiments were compared with standard antibiotics. All the statistical analysis was done by using IBM SPSS Statistics Data editor Version 20. The differences of means between the groups were analyzed using non-parametric test (Post hoc Tukey’s *t* test). A two sided p value of < 0.05 was considered as statistically significant.

## Results

### Antimicrobial screening of *Berberis aristata*

The aqueous extract of *Berberis aristata* root bark exhibited a broad spectrum antimicrobial potential ranging from 12 to 25 mm (Table 1). *Klebsiella pneumoniae* 1 was most susceptible organism (25 ± 0.889 mm) followed by *Staphylococcus aureus *> MRSA >* Salmonella typhimurium* 2 > *Staphylococcus epidermidis. Enterococcus faecalis* was found to be the least sensitive organism (12.75 ± 0.322 mm), whereas *Klebsiella pneumoniae* 2*, Shigella flexneri* and *Salmonella typhimurium* 1 were completely resistant to the extract. Of the two yeast cultures tested, *Candida albicans* exhibited an inhibition zone of 22.75 ± 0.595 mm while *Candida tropicalis* was completely resistant. It can also be depicted from Table 1 that the sensitivity of microorganisms was significantly different from each other. It was observed that the inhibition zone (in mm) of *Enterococcus faecalis* and *Escherichia coli* v/s *Candida albicans*, MRSA, *Klebsiella pneumoniae* 1, *Salmonella typhimurium* 2, *Staphylococcus epidermidis, Staphylococcus aureus, Pseudomonas aeruginosa* and of *Klebsiella pneumoniae* 1 v/s *Pseudomonas aeruginosa* showed a significant statistical differences (p ≤ 0.05), when compared with each other.

**Table 1:** Antimicrobial activity of 10% aqueous extract of *Berberis aristata* against some potential pathogens

Test-microorganism	Zone of inhibition (in mm)*
*Enterococcus faecalis*	12.75 ± 0.322^bdjklmn^
*Staphylococcus aureus*	22.62 ± 1.143^hm^
*Staphylococcus epidermidis*	21 ± 1.099^gl^
MRSA	21.75 ± 0.924^cd^
*Escherichia coli*	16 ± 0.912^acefgh^
*Klebsiella pneumoniae* 1	25 ± 0.889^eij^
*Klebsiella pneumoniae* 2	–
*Pseudomonas aeruginosa*	19.37 ± 0.826^in^
*Shigella flexneri*	–
*Salmonella typhimurium* 1	–
*Salmonella typhimurium* 2	21.25 ± 1.796^fk^
*Candida albicans*	22.75 ± 0.595^ab^
*Candida tropicalis*	–

–, No zone of inhibition

* The values are expressed as mean ± standard error of means (SEM) of four determinations. The values with the same letter in the superscript have a significant statistical difference (p ≤ 0.05) as indicated by Post hoc Tukey’s t-test

### Optimization of the parameters using classical approach

The antimicrobial activity gradually escalated with the increasing concentration up to 15% and thereafter only a marginal increase was noted. A significant antimicrobial potential was observed at an extraction temperature of 30 °C, which increased considerably up to 40 °C and thereafter declined insignificantly up to 100 °C. Upon extracting the plant material for different time intervals (20–240 min), the maximal antimicrobial activity was observed after extraction for 40 min. The extract suffered a considerable loss in activity at both acidic and alkaline pH, when compared to its activity at natural pH (4.5). The filtration through Whatman filter paper no. 1 showed significant activity, which marginally decreased in other tested methods. Therefore, 15% concentration prepared at 40 °C for 40 min were considered to be optimal conditions. Test organisms such as *Klebsiella pneumoniae* 2*, Shigella flexneri, Salmonella typhimurium* 1 and *Candida tropicalis* did not show any sensitivity under all the test conditions.

### Optimization of the physiochemical parameters using statistical method

#### Fitting the model and validation

The quadratic model was found to be a complete fit as the R^2^ ranged between 81.3 and 96.4% which showed suitable fitting of the model. The equations for each response: *S. aureus* (G_1_), *E. coli* (G_2_), *K. pneumoniae* 1 (G_3_), MRSA (G_4_) are given below as:

$${\text{G}}_{(1)} = 26.45+ 1.960{\text{X}}_{1} - 0.552 {\text{X}}_{2} - 0.375 {\text{X}}_{3} - 0.067 {\text{X}}_{1}^{2} + 0.0008 {\text{X}}_{2}^{2} + 0.003 {\text{X}}_{3}^{2} + 0.012 {\text{X}}_{1} {\text{X}}_{2} - 0.005 {\text{X}}_{1} {\text{X}}_{3} + 0.0062 {\text{X}}_{2} {\text{X}}_{3}$$

$${\text{G}}_{( 2)} = { 6}. 3 2 5+ \, 0. 1 10{\text{X}}_{ 1} - \, 0. 3 3 3 {\text{X}}_{ 2} + \, 0. 6 3 7 {\text{X}}_{ 3} - \, 0.0 4 7 {\text{X}}_{ 1}^{ 2} + \, 0.00 3 {\text{X}}_{ 2}^{ 2} - \, 0.00 9 {\text{X}}_{ 3}^{ 2} + \, 0.00 5 {\text{X}}_{ 1} {\text{X}}_{ 2} - + \, 0.0 20{\text{X}}_{ 1} {\text{X}}_{ 3} - \, 0.00 1 {\text{X}}_{ 2} {\text{X}}_{ 3}$$

$${\text{G}}_{( 3)} = { 55}. 7 7 5- \, 0. 7 30{\text{X}}_{ 1} - \, 0. 7 1 1 {\text{X}}_{ 2} - \, 0. 5 3 7 {\text{X}}_{ 3} - \, 0.0 4 9 {\text{X}}_{ 1}^{ 2} + \, 0.00 3 {\text{X}}_{ 2}^{ 2} + \, 0.000 2 {\text{X}}_{ 3}^{ 2} + \, 0.0 1 5 {\text{X}}_{ 1} {\text{X}}_{ 2} + \, 0.0 30{\text{X}}_{ 1} {\text{X}}_{ 3} + \, 0.00 3 {\text{X}}_{ 2} {\text{X}}_{ 3}$$

$${\text{G}}_{(4)} = 49.375 + 2.175 {\text{X}}_1 - 0.518 {\text{X}}_2 - 0.975 {\text{X}}_3 - 0. 10 5 {{\text{X}}_{1}^{ 2}} + 0.002 {{\text{X}}_{ 2}^{2}} + 0.006 {{\text{X}}_{3}^{2}} + 0.0 1 2 {\text{X}}_1 {\text{X}}_2 + 0.01 {\text{X}}_1 {\text{X}}_3 + 0.002 {\text{X}}_2 {\text{X}}_3 .$$

From the overall assessment, temperature of 45–55 °C, concentration: 15% and extraction time: 40–55 min can be considered as the optimized conditions for antimicrobial activity, which resulted into an enhanced antimicrobial activity by 1.26-folds for *S. aureus* (R^2^ = 96.4%), 1.13-folds (*E. coli*) (R^2^ = 86%), 1.17-folds (*K. pneumoniae* 1) (R^2^ = 81.3%) and 1.30-folds against MRSA (R^2^ = 92.4%).

#### Thermostability studies

One hour exposure of the aqueous extract to 60 °C resulted in 4.54–25% loss in antimicrobial activity against different organisms. At this temperature, the extract maintained its strong potency against MRSA, *Staphylococcus epidermidis*, *Enterococcus faecalis*, *Staphylococcus aureus* and *Candida albicans*, which was marginally lost (6.38–7.5%) at higher temperatures (70–90 °C). Exposure to the boiling temperature resulted in a maximum loss of only 40% (against *Pseudomonas aeruginosa*), however, the extract completely lost its activity against *Salmonella typhimurium* 2.

### Determining the best organic solvent for efficient extraction of antimicrobial metabolites

Ethyl acetate was the most suitable organic solvent for the maximum extraction of antimicrobial metabolites and exhibited an average inhibition zone of 25.46 mm. In this case, *Klebsiella pneumoniae* 1 showed the highest susceptibility (35.5 mm). The plant material extracted with other organic solvents were active in the order: Hexane (24.23 mm) > butanol (23.92 mm) > chloroform (21.38 mm). Both the yeast strains, i.e., *Candida albicans* (28 mm) and *Candida tropicalis* (22.5 mm) were notably sensitive to the plant material extracted with ethyl acetate.

### Qualitative and quantitative profiling of phytoconstituents

**Fig. 1:**
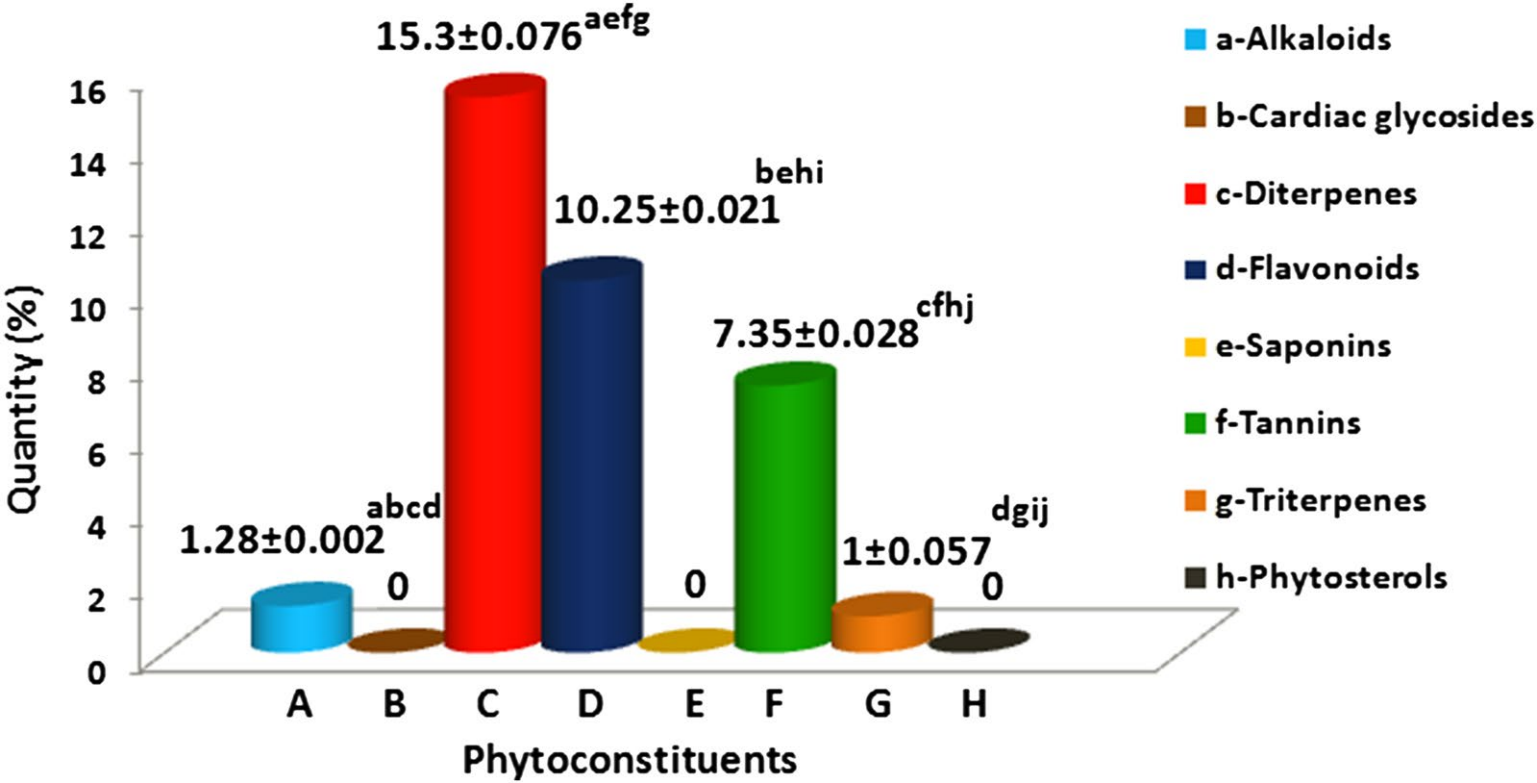
The concentrations of various phytoconstituents of *Berberis aristata* root bark (%/g). *The values are expressed as mean ± SEM for N = 3. **Same superscript alphabetic letters within the rows show significant statistical difference (p ≤ 0.05) among test organisms as indicated by Post hoc Tukey’s t-test

The major groups of phytoconstituents detected were alkaloids, flavonoids, triterpenes, diterpenes, anthranol glycosides, coumarins and tannins (Table 2), whereas saponins, cardiac glycosides and phytosterols were absent. In terms of yield (%/g), diterpenes were the most abundant (15.3%/g) followed by flavonoids (10.25%/g), alkaloids (1.28%/g) and triterpenes (1%/g) (Fig. 1).

**Table 2:** Qualitative detection and antimicrobial activity of *Berberis aristata* root bark

Phytoconstituents	Detected group	Stock solution (mg/mL)	Antimicrobial activity
***Alkaloids***		21.33	++^e^
Mayer’s reagent test	+		
Hager’s reagent test	+		
Wagner reagent test	+		
***Flavonoids***		68.33	+++^c^
Shinoda test (magnesium turnings)	+^b^		
Zinc-hydrochloride reduction test	+		
Lead acetate test	+		
Ferric chloride reagent test	−^a^		
***Saponins***		NA	NA
Froth test	−		
***Tannins***		73.5	−^f^
Ferric chloride reagent test	−		
Lead acetate test	+		
***Cardiac glycosides***		NA	NA
Keller–Killiani test	−		
***Terpenoids***			
Triterpenes (Salkowski’s test)	+	10	+^d^
Diterpenes (Copper acetate test)	+	87.42	+++^c^
***Anthranol glycosides***		ND	ND
Borntrager’s test	+		
***Phytosterols***		NA	NA
Libermann Burchard’s test	−		
Salkowski’s test	−		
***Coumarins***	+	ND	ND

NA, not applicable; ND, not done

^a^Absent

^b^Present

^c^Most active

^d^Least active

^e^Active

^f^Not active

Diterpenes were the most active group, exhibiting a broad spectrum antimicrobial potential against 12 out of the 13 test strains, with an inhibition zone ranging from 16.66 to 42.66 mm (Table 3). Among all the test strains, *Pseudomonas aeruginosa* was the most sensitive organism (42.66 mm). The antimicrobial potential of diterpenes was comparable to that of standard antibiotics. Flavonoids also exhibited a high potential activity against the 11 test strains which ranged from 13.66 to 35.6 mm, with *Klebsiella pneumoniae* 2 being the most susceptible one. Their activity was comparable to that of the standard antibiotics in most of the cases, but was better in case of *Staphylococcus aureus* and *Candida tropicalis*. Alkaloids were the third most active group with inhibition zone ranging from 12.33 to 23.3 mm. These three phytoconstituents were highly effective against *Candida albicans* (23.3 mm–42.33 mm), while only flavonoids and diterpenes were active against *Candida tropicalis*. Triterpenes were effective only against *Klebsiella pneumoniae* 1, while tannins were completely inactive against all the test organisms.

**Table 3:** Antimicrobial activity of phytoconstituents isolated from *Berberis aristata* root bark and its comparison to standard antibiotics (gentamicin, chloramphenicol and amphotericin B)

Test-microorganism^d^	Average zone of inhibition (mm)^b^			Gentamicin	Chloramphenicol
	Alkaloids	Flavonoids	Diterpenes		
SA	16.66 ± 0.333	29.33 ± 0.333	27.33 ± 0.333	34.5 ± 0.50	26 ± 1.00
SE	16.66 ± 0.333	16.33 ± 0.333	18.66 ± 0.333	26.5 ± 0.50	28.5 ± 0.50
EC	13.33 ± 0.333	17.66 ± 0.666	19.66 ± 0.333	31 ± 1.00	25 ± 0
EF	12.33 ± 0.333	24.66 ± 0.333	29.66 ± 0.333	27.5 ± 0.50	26.5 ± 0.50
KP1	21.33 ± 0.333	31.3 ± 0.333	35.66 ± 0.333	40.5 ± 0.50	38 ± 1.00
KP2	21 ± 0	35.6 ± 0.333	35.33 ± 0.333	37.5 ± 1.50	26.5 ± 0.50
SF	0^a^	0	16.66 ± 0.333	30.5 ± 0.50	27.5 ± 0.50
ST1	0	0	0	35 ± 0	23 ± 0
ST2	16.66 ± 0.333	30.33 ± 0.333	27.33 ± 0.333	43 ± 1.00	40.5 ± 0.50
PA	14.33 ± 0.333	13.66 ± 0.333	42.66 ± 0.333	40.5 ± 0.50	28.5 ± 0.50
CA	23.3 ± 0.666	33.66 ± 0.333	42.33 ± 0.333	36.5 ± 0.50^c^	ND
CT	0	30 ± 0.577	30.66 ± 0.333	27.5 ± 0. 50^c^	ND
MRSA	15 ± 0	28.33 ± 0.666	36.33 ± 0.333	42 ± 0	39.5 ± 0.50

ND, not done

^a^No activity

^b^Values are expressed as mean ± standard error of means (SEM) of three determinations

^c^Amphotericin B

^d^Test-microorganism—SA, *Staphylococcus aureus*; SE, *Staphylococcus epidermidis*; EC, *Escherichia coli*; KP1, *Klebsiella pneumoniae* 1; KP2, *Klebsiella pneumoniae* 2; SF, *Shigella flexneri*; ST1, *Salmonella typhimurium* 1; ST2, *Salmonella typhimurium* 2; PA, *Pseudomonas aeruginosa*; CA, *Candida albicans*; CT, *Candida tropicalis*; MRSA, methicillin-resistant *Staphylococcus aureus*

### Minimum inhibitory concentration (MIC)

The MIC values obtained for the test extracts were organism-specific (Table 4). For the aqueous extract, it ranged from 2.5 to 25 mg/mL. Ethyl acetate extract was highly potent and exhibited a very low MIC range (0.05–1 mg/mL). Among the partially purified phytoconstituents (PPPs), diterpenes were the most active with MIC values ranging from 0.05 to 5 mg/mL. Flavonoids were effective in the range of 0.05 to 10 mg/mL whereas alkaloids exhibited the MIC values varying from 0.7 to 10 mg/mL.

**Table 4:** Minimum inhibitory concentration (MIC) of *Berberis aristata* extracts, its partially purified phytoconstituents (PPPs) and standard antibiotics

Microorganisms	MIC (mg/mL)						
	Aq^a^	EA^b^	Alkaloids	Flavonoids	Diterpenes	Gentamicin	Chloramphenicol
SA	2.5	0.05	1	0.1	0.5	0.0002	0.01
SE	25	0.1	3	3	3	0.01	0.01
MRSA	5	0.05	5	0.1	0.05	0.005	0.01
EC	5	0.05	5	3	3	0.005	0.01
EF	22.5	0.1	ND	1	1	0.03	0.3
KP1	2.5	0.05	1	0.05	0.1	0.0002	0.01
KP2	ND	0.1	1	0.5	0.7	0.0005	0.001
SF	ND	0.05	ND	ND	5	0.005	0.01
ST1	ND	0.05	ND	ND	ND	0.005	0.1
ST2	15	0.05	1	0.05	0.1	0.0003	0.001
PA	5	0.05	10	10	0.05	0.005	0.7
CA	7.5	0.05	0.7	0.1	0.05	0.0003^c^	ND
CT	ND	0.5	ND	1	1	0.1^c^	ND

SA, *Staphylococcus aureus*; SE, *Staphylococcus epidermidis*; EC, *Escherichia coli*; KP1, *Klebsiella pneumoniae* 1; KP2, *Klebsiella pneumoniae* 2; SF, *Shigella flexneri*; ST1, *Salmonella typhimurium* 1; ST2, *Salmonella typhimurium* 2; PA, *Pseudomonas aeruginosa*; CA, *Candida albicans*; CT, *Candida tropicalis*; MRSA, methicillin-resistant *Staphylococcus aureus*; ND, not determined

^a^Aqueous extract

^b^Ethyl acetate extract

^c^Amphotericin B

### Viable cell count studies

**Fig. 2:**
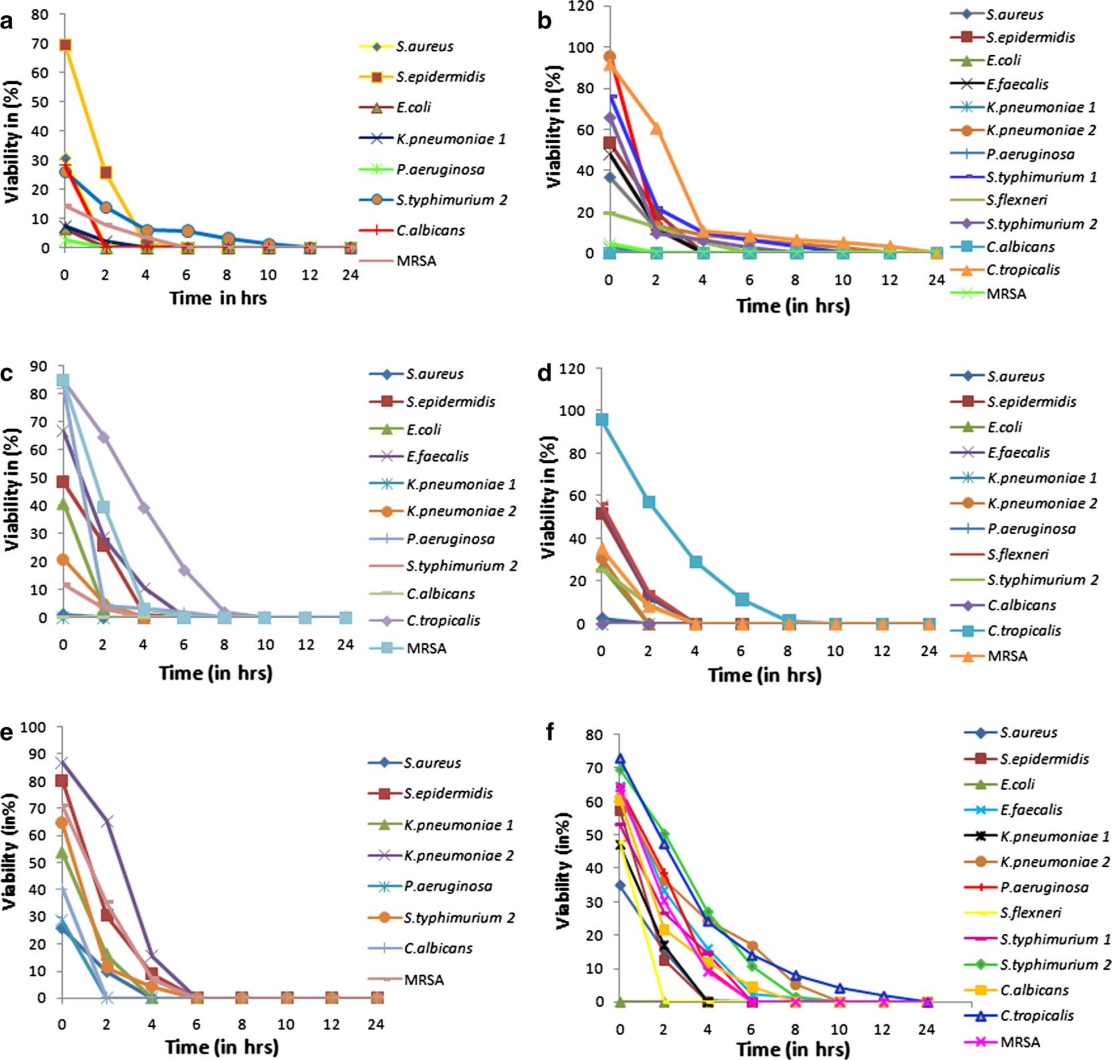
Viable cell count (VCC) studies for (**a**) aqueous extract, (**b**) organic extract, (**c**) flavonoids, (**d**) diterpenes, (**e**) alkaloids of *Berberis aristata* root bark and (**f**) standard antibiotic Gentamicin (*Amphotericin B for yeast strains). SA, *Staphylococcus aureus*; SE, *Staphylococcus epidermidis*; EC, *Escherichia coli*; KP1, *Klebsiella pneumoniae* 1; KP2, *Klebsiella pneumoniae* 2; SF, *Shigella flexneri*; ST1, *Salmonella typhimurium* 1; ST2, *Salmonella typhimurium* 2; PA, *Pseudomonas aeruginosa*; CA, *Candida albicans*; CT, *Candida tropicalis*; MRSA, methicillin-resistant *Staphylococcus aureus*

The aqueous extract was effective against most of the test organisms with the killing time ranging from 2 to 12 h. The ethyl acetate extract was highly potent, as killing time was similar to that of standard antibiotics in case of organisms like *Staphylococcus epidermidis, Escherichia coli* and *Candida tropicalis*. Flavonoids and diterpenes were highly effective and instantaneously killed *Klebsiella pneumoniae* 1, *Candida albicans* and *Pseudomonas aeruginosa*. The kill time studies revealed that the PPPs were more active against the yeasts *Candida albicans* (0 h) and *Candida tropicalis* (10 h) as compared to the standard antibiotic amphotericin B, which required 8 h and 24 h respectively for complete killing (Fig. 2).

### Determination of the post antibiotic effect of the ethyl acetate extract, flavonoids and diterpenes

**Fig. 3:**
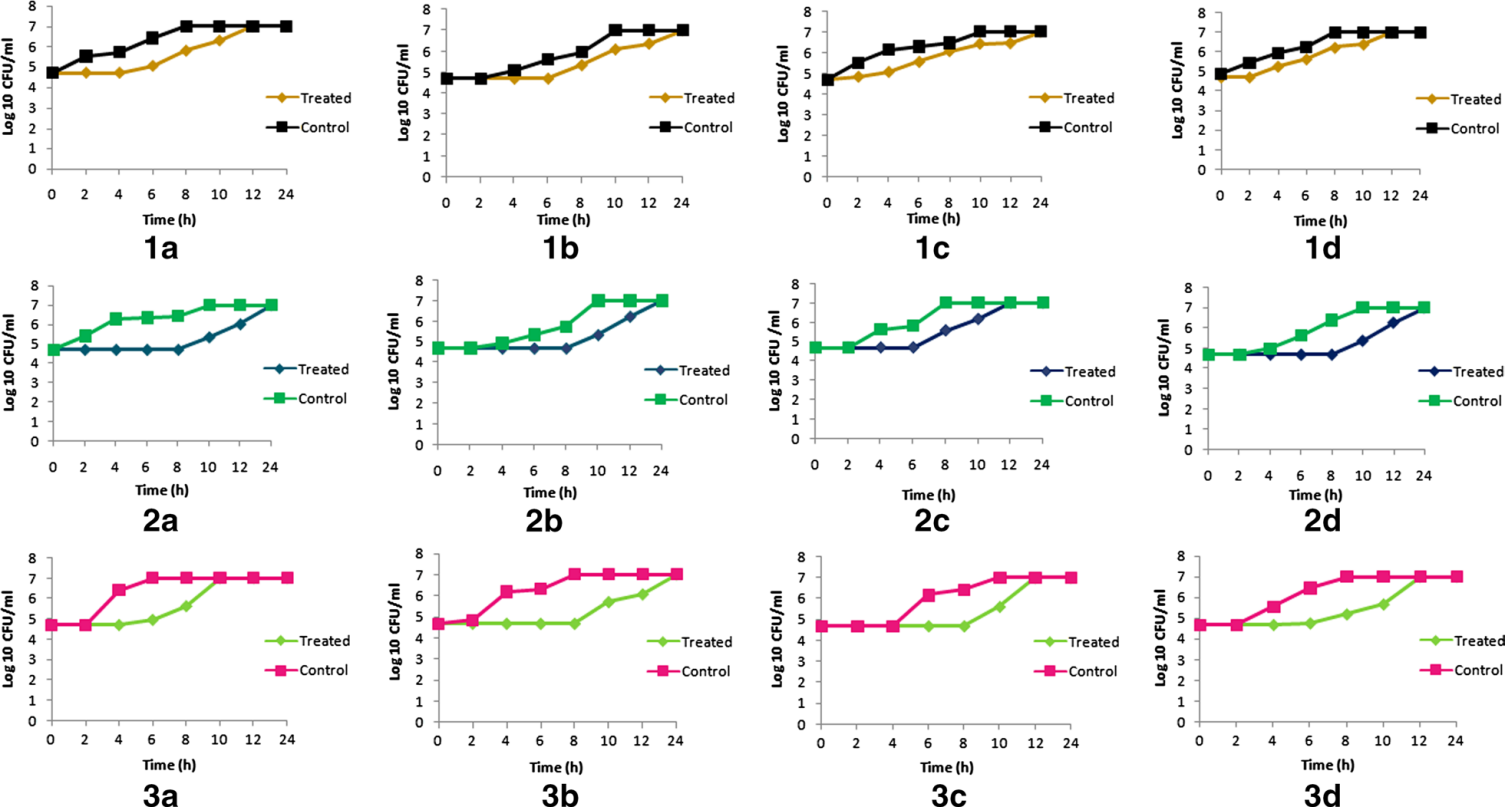
Post antibiotic effect (PAE) of (1) ethyl acetate extract (2) diterpenes (3) flavonoids of *Berberis aristata* root bark against (**a**) *Staphylococcus aureus,* (**b**) *Salmonella typhimurium* 2, (**c**) *Klebsiella pneumoniae* 2, (**d**) *Candida albicans*

The PAE of the ethyl acetate extract ranged from 2 to 4 h, whereas for phytoconstituents it varied from 4 to 8 h (Fig. 3). The ethyl acetate extract of *Berberis aristata* was least effective against *Klebsiella pneumoniae* 2 and *Candida albicans* with a PAE of 2 h whereas it was 4 h against both *Staphylococcus aureus* and *Salmonella typhimurium* 2. Among PPPs, diterpenes were the most potent with a prolonged effect up to 8 h whereas flavonoids exhibited maximum effect up to 6 h. In case of *Candida albicans*, diterpenes were more effective (6 h) than the flavonoids (4 h).

### Biosafety evaluation

All the test extracts were evaluated for their ability to cause mutagenicity and cytotoxicity. In the Ames test, the aqueous extract showed 28 revertant colonies in comparison to the sodium azide (758 colonies), whereas no revertant colonies were observed for the organic extract and phytoconstituents when compared to the sodium azide (856 colonies). Hence, the test extracts were considered to be non-mutagenic. In MTT assay, the test extracts showed % viability of 88.09% (aqueous extract), 86.10% (organic extract), 89.17% (alkaloids), 91.49% (flavonoids) and 89.94% (diterpenes), thus indicating their non-cytotoxic nature.

### In vitro cytotoxicity by MTT assay

**Fig. 4:**
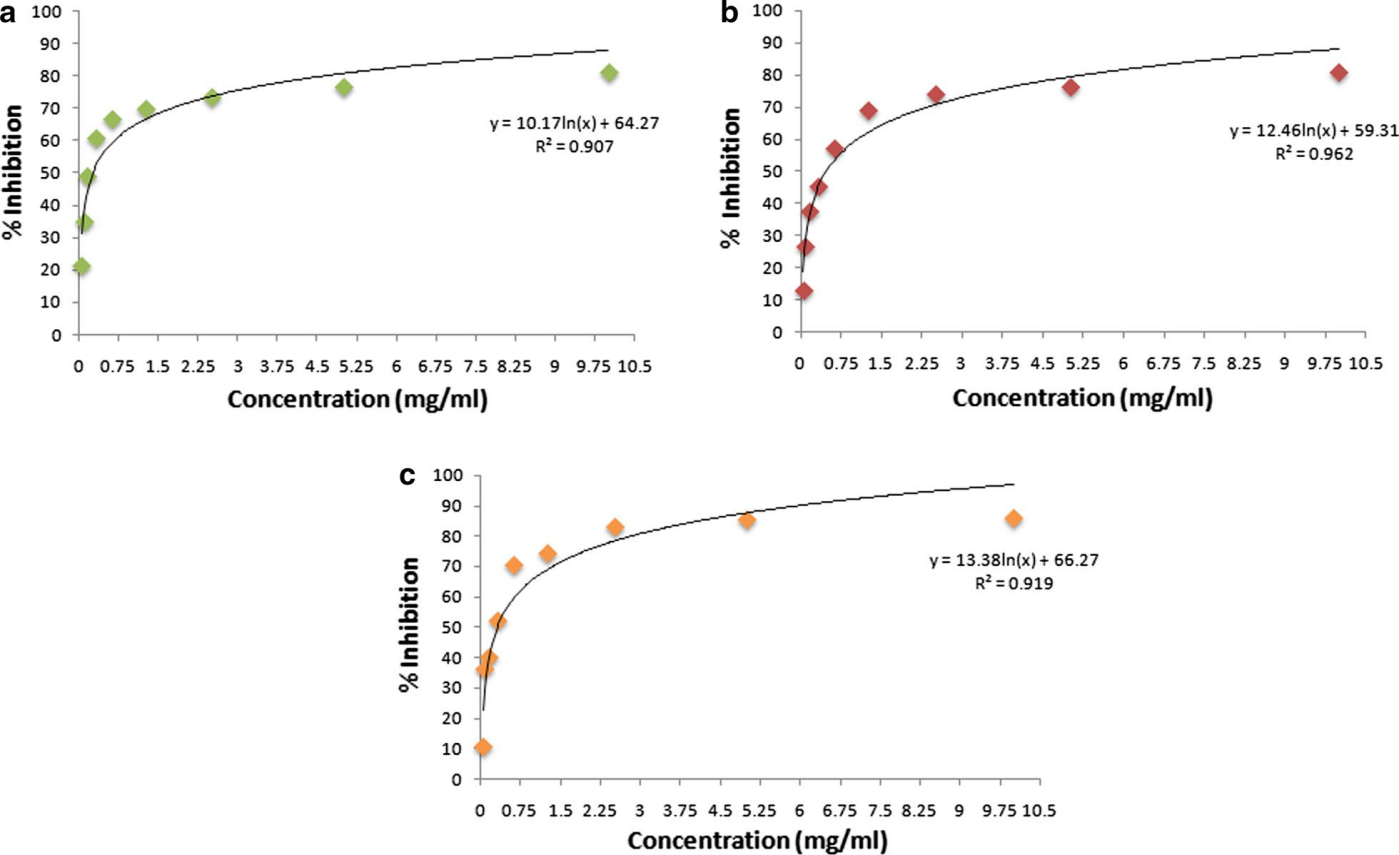
*In vitro* cytotoxicity of *Berberis aristata* root bark on (**a**) RD, (**b**) L20B, (**c**) Hep 2 cell line

The most active phytoconstituent of *Berberis aristata*, i.e., diterpenes, when tested against three cell lines (L20B, RD and Hep2), displayed a good cytotoxic effect with IC_50_ values ranging from 245 to 473 µg/mL. The effect was best seen against RD cell line [IC_50_ = 245 µg/mL], where even the lowest concentration (0.039 mg/mL) showed 21.58% inhibition. In case of Hep 2 cell line, the diterpenes exhibited an IC_50_ of 296 µg/mL whereas against L20B, a value of 473 µg/mL was obtained (Fig. 4).

### Acute oral toxicity study of the *Berberis aristata* diterpenes on Swiss albino mice

**Fig. 5:**
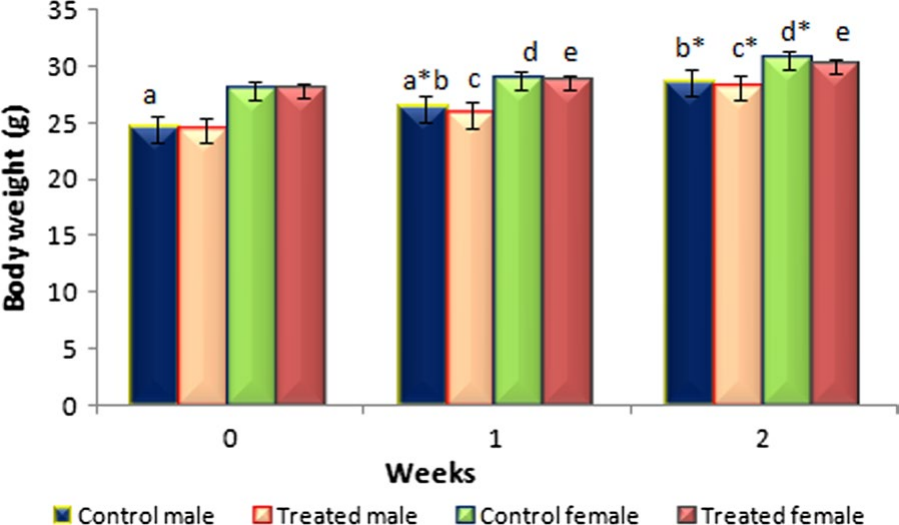
Mean body weight of mice upon dosage of *Berberis aristata* diterpenes. Values are expressed as mean ± SEM (n = 6 for each group). Same superscript alphabetic letters above the bars show significant statistical difference. Asterisk denotes (p ≤ 0.05) as indicated by Post hoc Tukey’s t-test

**Fig. 6:**
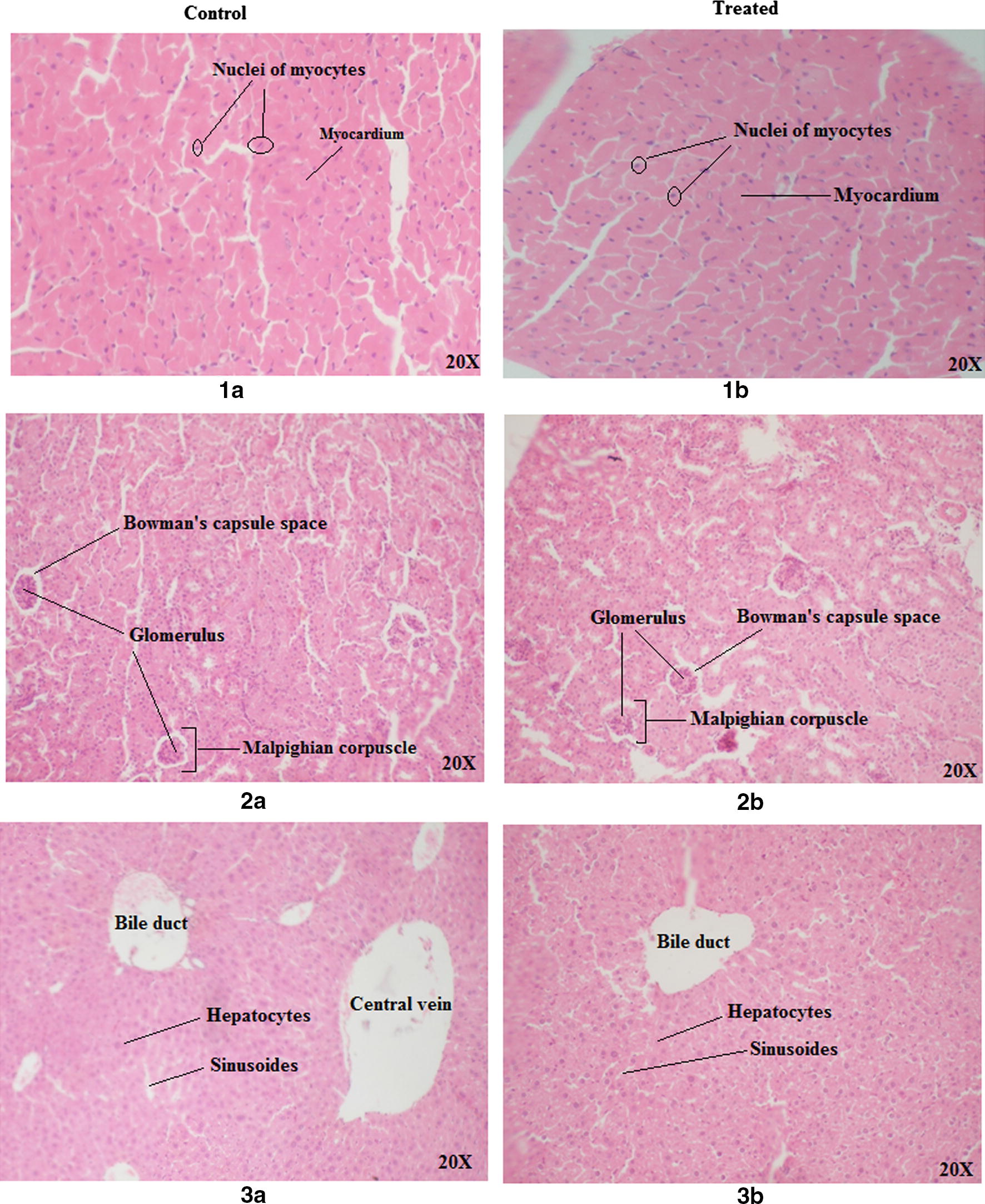
Photomicrographs of the tissue sections of (**a**) heart (**b**) kidney and (**c**) liver taken from mice treated with *Berberis aristata* diterpenes for 14 days for assessment of acute oral toxicity in comparison with untreated control

*Berberis aristata* diterpenes, at a dose of 5000 mg/kg b.wt, showed no adverse effect on the responses of the tested mice up to 14 days of observation. No signs or changes were observed in behavioral and movement patterns, breathing, skin, fur, eyes, salivation and sleep. Tremors, diarrhea and lethargy were also not observed. No weight loss was seen in all the mice (test and control), instead they exhibited a normal weight gain without any significant difference between the test and the control group (Fig. 5). No significant difference was observed in the absolute and relative organ weight of the liver, kidney and heart of the test groups (male and female) when compared with the control group. In this study, no significant changes in the serum levels of kidney function parameters (Urea, Creatinine) and liver function parameters [total bilirubin, aspartate aminotransferase (AST), alanine aminotransferase (ALT), alkaline phosphatase (ALP)] were seen for the treated groups in comparison to the control groups (Tables 5, 6). Macroscopic examination of the three vital organs revealed no abnormalities in the color or texture. No histopathological changes were observed in comparison to the control in any of the tissues examined (Fig. 6). The heart, kidney and liver showed normal architecture and cellular details of the myocardium, glomeruli-tubules and hepatocytes, respectively.

**Table 5:** Absolute and relative organ weight of control and treated mice (male and female) in the acute toxicity study of *Berberis aristata* diterpenes

Organs	Absolute organ weight (g)*		Relative organ weight (%)*	
	Control	Treated	Control	Treated
***Male***				
Liver	0.724 ± 0.031	0.716 ± 0.031	2.453 ± 0.031	2.442 ± 0.030
Kidneys	0.401 ± 0.022	0.392 ± 0.023	1.355 ± 0.035	1.333 ± 0.034
Heart	0.165 ± 0.007	0.160 ± 0.007	0.558 ± 0.007	0.546 ± 0.009
***Female***				
Liver	0.827 ± 0.044	0.813 ± 0.031	2.613 ± 0.055	2.633 ± 0.048
Kidneys	0.410 ± 0.023	0.399 ± 0.016	1.297 ± 0.0353	1.294 ± 0.026
Heart	0.166 ± 0.0118	0.159 ± 0.0062	0.524 ± 0.0195	0.516 ± 0.0104

* Data indicate mean ± SEM (n = 6 for each group). There was no significant difference (p > 0.05) between the test and control groups as indicated by Post hoc Tukey’s t-test

**Table 6:** Effect of *Berberis aristata* diterpenes on biochemical parameters in acute oral toxicity study

Parameters	Male		Female	
	Control*	Treated*	Control*	Treated*
Urea (Mmol/L)	42.633 ± 0.839	42.45 ± 0.476	40.13 ± 0.991	37.933 ± 0.987
Creatinine (µmol/L)	0.774 ± 0.026	0.770 ± 0.009	0.618 ± 0.024	0.566 ± 0.008
Total bilirubin (µmol/L)	2.376 ± 0.093	2.348 ± 0.014	1.76 ± 0.025	1.695 ± 0.015
Aspartate aminotransferase (AST) (U/L)	208.96 ± 4.192	212.13 ± 0.705	160.66 ± 2.708	162.08 ± 0.351
Alanine aminotransferase (ALT) (U/L)	79.95 ± 2.095	77.80 ± 0.566	61.33 ± 2.677	64.186 ± 0.520
Alkaline phosphatase (ALP) (U/L)	258.3 ± 2.510	260.3 ± 0.813	183.33 ± 1.612	185.13 ± 0.305

* Values are expressed as mean ± SEM (n = 6 for each group). There was no significant difference (p > 0.05) between the test and control groups as indicated by Post hoc Tukey’s t-test

## Discussion

There has been a continuous struggle between the mankind and the infectious pathogens for proving their supremacy. Evaluation of natural products as safe and effective antimicrobial agents can be used as a scientific strategy to overcome the nuisance caused by drug-resistant pathogens. Hence, a systematic and scientific screening of medicinal plants could provide an answer to the problem (Al-Daihan et al. 2013). Keeping this in mind the study has been directed towards providing scientific proof to the traditional use of one such medicinal plant.

The preliminary screening of *Berberis aristata* aqueous extract revealed its broad spectrum antimicrobial activity against various potential pathogens including *Pseudomonas aeruginosa, Staphylococcus aureus, Klebsiella pneumoniae* 1 and *Escherichia coli* which is in line with previous studies on other medicinal plants (Chandra 2013; Khan et al. 2013). The plant was quite effective against *Candida albicans* and drug-resistant organisms such as methicillin-resistant *Staphylococcus aureus* (MRSA). Further, in the classical optimization of the physiochemical parameters, 15% concentration was found to be optimal which is in concurrence with earlier reports, where 10–20% concentration was effective (Al-Sum and Al-Arfaj 2013; Abuzied et al. 2014). Similarly, optimal extraction temperature (40 °C) is in line with Arora and Mahajan (2018). The optimal extraction time (40 min) and the natural pH (4) go well with similar studies where 30 min extraction at natural pH (5.0) was optimal (Dent et al. 2013). The relatively higher stability of the active compound in its natural environment may be the reason behind higher potency of the extract at unadjusted pH. The filtration through the Whatman filter paper was best, which may be attributed to the optimal pore size required for the active molecules to pass through. The statistical optimization of these parameters resulted in an overall increase in antimicrobial potential by 1.13–1.3-folds, which is comparable to values obtained earlier (Arora and Mahajan 2018; Rajeswari et al. 2010). Relatively good thermostability of the aqueous extract at boiling temperature may prove quite helpful when exploiting the plant material for commercial purposes. Among the organic solvents, ethyl acetate was the best, which may be owing to the better solubility of the bioactive molecules in this solvent as compared to other solvents. Qualitative testing demonstrated the presence of major phytoconstituent groups except the saponins, cardiac glycosides and phytosterols, which is in line with earlier studies (Arora and Onsare 2014a; Sharma and Sharma 2015). Among the detected phytoconstituents, diterpenes were not only the most abundant but also the best antimicrobials, in consonance with a similar study on *Moringa oleifera* seed coat (Monte et al. 2014). The MIC values of the test extracts well support the agar diffusion assay (ADA) results. For example, in case of diterpenes, *Escherichia coli* and *Staphylococcus epidermidis* were the least sensitive organisms (18–19 mm), which corresponded well with their highest MIC (3 mg/mL) among all test organisms. Similarly, *Pseudomonas aeruginosa* was the most sensitive organism (42.66 mm) and showed the lowest MIC among the test organisms (0.05 mg/mL). In case of yeast, *Candida albicans* was more sensitive to the test extracts (0.05–0.1 mg/mL) than *Candida tropicalis* (0.5–1 mg/mL). Ethyl acetate extract was highly effective against the two yeasts, followed by diterpenes and flavonoids. The study got more credence with the observation that in case of *Pseudomonas aeruginosa,* alkaloids and flavonoids showed the highest MIC value (10 mg/mL) among all the test extracts whereas in case of resistant strains like MRSA, the different test extracts were quite effective in a concentration range of 0.05–5 mg/mL. The results obtained were comparable to the MIC range of 0.2–3.2 mg/mL obtained for various phytochemicals against *E. coli* and *S. aureus* (Awe and Amobi 2015). It was found that the MIC values of all test extracts were much lower than values (300–350 mg/mL and 12.5–25 mg/mL) obtained in an earlier report on *Pteridium aquilinum* hexane extract and various ethiopian medicinal plants, respectively (Bacha et al. 2016; Elisha et al. 2017), which highlights the importance of this medicinal plant. The total activity potency (TAP) reflects the efficacy of the extracts which provided the scientific validation for the selection of *Berberis aristata* as a medicinal plant. The TAP range for phytoconstituents was from 1.28 to 3060 mL/g, which was comparable or better than the earlier observation for different plant species against various pathogens (Arora and Onsare 2014b).

Instantaneous killing of the potential pathogens like *P. aeruginosa* and *C. albicans* by the organic extract, flavonoids and diterpenes provide credence to the study. The importance of the study is also highlighted as the organic extract and PPPs have effectiveness comparable to standard antibiotics in case of some bacteria and yeast strains, respectively. The post antibiotic effect (PAE), usually used to develop the antimicrobial dosing regimens on a scientific basis demonstrated varied duration of PAE against different microbes for different test extracts. The organic extract exhibited a PAE of 2–4 h which was much longer than obtained for *Moringa oleifera* stem bark (0.6–3.3 h) (Harput et al. 2011). The PAE of PPPs was much longer (4–10 h) in comparison to the organic extract, which highlight their importance as natural antimicrobials. Further, it is important for any compound to be completely biosafe so as to prove its candidature as a potential drug for further use. In the present study, the test extracts were completely biosafe as they were adjudged as non-cytotoxic and non-mutagenic according to the Ames and MTT assay. This study holds more significance as the use of natural products as cytotoxic agents against cancerous cells has a long history in folk medicine. In the present study, the most active phytoconstituents (diterpenes) was also tested for its cytotoxic effect against three cell lines (Hep 2, RD and L20B). The IC_50_ values obtained were much lower than those obtained for the *Acanthus hirsutus*, *Veronica cuneifolia* subsp. *cuneifolia*, *Veronica cymbalaria* and *Nicotiana tabacum* extracts in some similar reports on Hep 2, RD and L20B cell lines (Saracoglu et al. 2011; Al-Asady et al. 2014; Malik et al. 2017).

To add further credence to the study, toxicity of the diterpenes was checked using acute oral toxicity study in mice. This aspect is crucially important as toxicity results from animals help in judging the safety of the compounds if they are found to have sufficient potential for development into pharmacological products (Jothy et al. 2011). In this study, the biosafety of the diterpenes have been demonstrated using mice model as no symptoms of toxicity or death was observed, which means the compound did not adversely affect the metabolism and growth of the mice. The evaluation of various biochemical parameters also demonstrated the non-toxic nature of *Berberis aristata* diterpenes. The usage of this plant in traditional medicine gets further scientific validation from the histopathological analysis, as no pathological changes in any tissue were observed within the test and control group, which is in consonance with similar studies on other plant extracts, that were found to be non-toxic to test animals (Jothy et al. 2011; Ping et al. 2013; Murbach et al. 2014). However, in certain studies, the extracts have shown to slightly affect the organ morphology (Alade et al. 2009; Harizal et al. 2010). These finding highlight the importance of the study as the biosafe profile of the *Berberis aristata* extracts make it a suitable candidate for development of potential antimicrobial compounds in the time to come.

This study, thus, highlights the true potential of an important medicinal plant, i.e., *Berberis aristata* root bark, which exhibited a broad spectrum antimicrobial potential against potent human pathogens. Its test extracts were highly potent against *Pseudomonas aeruginosa, Candida albicans*, MRSA to name a few. They exhibited quite low MIC values and killing time and displayed a prolonged post antibiotic effect. Apart from being such potent molecules, they were found to be biosafe. Thus, its extracts and phytoconstituents, upon further purification, could add another molecule/s to the arsenal of novel metabolites.

## Supplementary information

**Additional file 1.** The standard methods for qualitative and quantitative estimation of the major group of phytoconstituents.

**Additional file 2.** In vitro cytotoxicity study using RD, L20B and Hep2 cell lines by MTT assay-culture medium, stock cultures, test dilutions and determination of cytotoxicity by MTT assay.

**Additional file 3.** Acute Oral Toxicity study of *Berberis aristata* diterpenes in Swiss albino mice-target animals, toxicity assay procedure (biochemical parameters, organ and body weight analysis, histopathological analysis).

## Data Availability

Not applicable.

## Abbreviations

ADA: agar diffusion assay
MTT: [3-(4,5-dimethylthiazol-2-yl)-2,5-diphenyl tetrazolium bromide]
MRSA: methicillin-resistant *Staphylococcus aureus*
MIC: minimum inhibitory concentration
PPPs: partially purified phytoconstituents
RSM: response surface methodology
TAP: total activity potency
MTCC: microbial type culture collection
VCC: viable cell count study
PAE: post antibiotic effect
