# Supplementary material for: Scientific validation of the antimicrobial and antiproliferative potential of Berberis aristata DC root bark, its phytoconstituents and their biosafety
Source: AMB Express. 2019 Sep 11;9:143. doi: 10.1186/s13568-019-0868-4 (PMC6738363; doi:10.1186/s13568-019-0868-4)
Supplement: Supplementary file 1 — Additional file 1. The standard methods for qualitative and quantitative estimation of the major group of phytoconstituents. [file 13568_2019_868_MOESM1_ESM.docx]

**Scientific validation of the antimicrobial potential of *Berberis aristata* DC root bark, its phytoconstituents and their biosafety in terms of Ames test, MTT assay and Acute Oral Toxicity study**

**Henna Sood^a^, Yashwant Kumar^b^, Vipan Kumar Gupta^c^ and Daljit Singh Arora^a^***

^a^ Microbial Technology Laboratory, Department of Microbiology, Guru Nanak Dev University, Amritsar-143005, India.

^b^ National Salmonella & Escherichia Centre and Diagnostic Reagents Laboratory, Central Research Institute, Kasauli (H.P.) – 173204, India.

^c^ Department of Veterinary Pathology, Dr. G.C. Negi College of Veterinary and Animal Sciences, CSK Himachal Pradesh Krishi Vishvavidyalaya, Palampur (H.P.) - 176062, India.

Tel. No. 91-183-2258802-09 Ext. 3506, Fax No. 91-183-2258819-20

*Corresponding author: Prof. Daljit Singh Arora, Microbial Technology Laboratory, Department of Microbiology, Guru Nanak Dev University, Amritsar-143005, India

1. **Qualitative analysis for the detection of major group of phytoconstituents**

Qualitative analysis was carried out using the powdered plant material by standard chemical methods as protocols mentioned in (Arora and Sood, 2017; Arora and Onsare, 2014c; Ezeonu et al., 2016; Kaur and Arora, 2009) and are described below:

**Alkaloids**: They were detected using Wagner’s, Meyer’s and Hager’s reagents separately. The tests were scored positive on the basis of brown precipitates, yellow precipitates and turbidity respectively. **Flavonoids**: Occurrence of pink or magenta red coloration, magenta coloration, bulky white precipitate and dark green precipitate, respectively in Shinoda test, Zinc–hydrochloride reduction, lead acetate and ferric chloride tests was considered as a positive result. **Saponins**: Formation of froth upon vigorous shaking indicated a positive test. **Tannins**: Appearance of brownish–green/blue–black coloration and gelatinous/bulky white precipitation in ferric chloride test and lead acetate test respectively is a positive indication. **Cardiac glycosides**: These were detected using Keller Killiani test, where formation of reddish brown ring at the interface indicated their presence. **Terpenoids**: Presence of triterpenes was detected using Salkowski’s test, with Golden yellow coloration as positive indication. Diterpenes were detected using copper acetate test, where formation of emerald green color indicated positive. **Anthranol glycosides:** 0.2 g of plant powder was suspended in 8  ml of 1  M HCl and hydrolyzed for 2h. Treatment of 2 ml of the hydrolysate with 5% ferric chloride solution, then an equal volume of benzene, which was then separated and treated with 10% ammonium solution, determined a formation of rose pink in ammonical layer in a positive case. **Phytosterols**: Were detected using Libermann Burchard’s test and Salkowski test. **Coumarins** Their presence was assayed by adding 3 ml of 10% NaOH to 2 ml of the aqueous extract, where formation of yellow color indicated positive test.

1. **Quantitative isolation of the detected phytoconstituents**

The phytoconstituents which were qualitatively detected in the plant material were quantitatively isolated by standard methods as per protocols given in (Arora and Sood, 2017; Arora and Onsare, 2014c; Ezeonu et al., 2016; Kaur and Arora, 2009)and are described below:

**Flavonoids:** Two gram of powdered plant material was extracted with 40ml of 80% aqueous methanol under shaking conditions for 24h at 30°C. The filtrate, thus obtained, was evaporated on a rotary evaporator and the residual dry weight was determined as flavonoids. **Saponins**: Ten gram plant powder was extracted in 50ml of 20% aqueous ethanol for 30min under shaking condition, and was thereafter kept in a water bath for 4h at 55°C. The filtrate obtained was reduced to 20 ml volume in a water bath at 90 °C, followed by three times extraction with 20ml of diethyl ether. The aqueous portion was retained, pooled and extracted twice with 60ml butanol. The butanolic layer was pooled and washed two times with 10ml of 5% NaCl. The butanolic portion was then concentrated and dried to obtain a constant dry weight of saponins. **Cardiac glycosides:** Plant powder (2g) was extracted three times with 40 ml of methanol under shaking conditions at 30°C for 24hrs. The combined filtrates were evaporated to dryness and then defatted with petroleum ether for 24hrs. Following decantation, the dried residue was redissolved in 50% aqueous methanol and was extracted three times with chloroform. The filtrates were pooled and concentrated to obtain the dry weight as cardiac glycosides. **Diterpenes:** Two gram of the plant powder was extracted three times in 40ml of 50% ethanol at 30 °C for 24h. The collected filtrates were pooled and evaporated to dryness. The dried residue was weighed as diterpenes. **Tannins:** For isolation of tannins, 2g of plant powder was extracted five times with a mixture containing 10ml of 8% sodium carbonate (Na_2_CO_3_) and 20 ml distilled water in the ratio 1:15 (w/v). A 10ml volume of HCl and 20ml formaldehyde was added to the combined filtrate and kept under reflux for 30 min. The mixture was filtered in a pre weighed Whatmann paper**,** which was oven-dried to obtain a constant weight as tannins. **Phytosterols:** The isolation was carried out according to Samria and Sarin (2014) with slight modifications. Here, 2g of powdered plant material was defatted three times in petroleum ether for 24h on a water bath. The defatted material was air-dried and hydrolyzed with 50ml of 30% HCl (v/v) for 4h. The sample was repeatedly washed with distilled water till pH 7 was obtained. The sample was then dried and then extracted three times with 40ml of benzene for 24h. The filtrates were combined and dried *in vacuo* to obtain the dried mass as phytosterols
