# Supplementary material for: Scientific validation of the antimicrobial and antiproliferative potential of Berberis aristata DC root bark, its phytoconstituents and their biosafety
Source: AMB Express. 2019 Sep 11;9:143. doi: 10.1186/s13568-019-0868-4 (PMC6738363; doi:10.1186/s13568-019-0868-4)
Supplement: Supplementary file 2 — Additional file 2. In vitro cytotoxicity study using RD, L20B and Hep2 cell lines by MTT assay-culture medium, stock cultures, test dilutions and determination of cytotoxicity by MTT assay. [file 13568_2019_868_MOESM2_ESM.docx]

**Scientific validation of the antimicrobial potential of *Berberis aristata* DC root bark, its phytoconstituents and their biosafety in terms of Ames test, MTT assay and Acute Oral Toxicity study**

**Henna Sood^a^, Yashwant Kumar^b^, Vipan Kumar Gupta^c^ and Daljit Singh Arora^a^***

^a^ Microbial Technology Laboratory, Department of Microbiology, Guru Nanak Dev University, Amritsar-143005, India.

^b^ National Salmonella & Escherichia Centre and Diagnostic Reagents Laboratory, Central Research Institute, Kasauli (H.P.) – 173204, India.

^c^ Department of Veterinary Pathology, Dr. G.C. Negi College of Veterinary and Animal Sciences, CSK Himachal Pradesh Krishi Vishvavidyalaya, Palampur (H.P.) - 176062, India.

Tel. No. 91-183-2258802-09 Ext. 3506, Fax No. 91-183-2258819-20

*Corresponding author: Prof. Daljit Singh Arora, Microbial Technology Laboratory, Department of Microbiology, Guru Nanak Dev University, Amritsar-143005, India

***In vitro* cytotoxicity study using RD, L20B and Hep2 cell lines by MTT assay**

**Culture medium**

Stock cultures were prepared in a 25cm^2^ tissue culture bottle containing Dulbecco’s Modified Eagle’s Medium (DMEM) supplemented with 10% inactivated Fetal Bovine Serum (FBS), penicillin (100 IU/ml), streptomycin (100 µg/ml) and amphotericin B (5 µg/ml) at 37 °C in a humidified atmosphere (90% RH) and 5% CO_2_ level.

**Standardization of stock cultures**

The cell line monolayer in each 25cm^2^ bottle was trypsinized using 2 ml TPVG solution (0.2% trypsin, 0.05% glucose and 0.02% EDTA in PBS). The Trypsin Phosphate Versene Glucose (TPVG) solution was then carefully discarded and a cell suspension was prepared using 2ml of DMEM with 10% FBS. The cell count in this suspension was adjusted to 1x10^5^ cells/ ml with DMEM (containing 10% FBS) by trypan blue dye exclusion technique using neubauer chamber.

**Preparation of test dilutions**

For the cytotoxic studies, a weighed quantity of *Berberis aristata* diterpene was dissolved separately in a known volume of DMSO. A stock solution (10mg/ml) was prepared from it using DMEM supplemented with 2% inactivated FBS and sterilized by filtration (0.2 µm syringe filter). Two fold serial dilutions ranging from 10mg/ml to 0.039mg/ml were prepared from this stock solution and were used in the experimentation.

**Determination of cytotoxicity by MTT assay**

Hundred microlitre (100 µl) of the diluted cell suspension (approx. 10,000 cells) of each cell line was added to a separate 96 well microtitre plate and incubated in a CO_2_ incubator at 37°C containing 5% CO_2_ for 24h. After 24hrs, the supernatant was removed and the monolayer was gently washed with the medium containing 10% FBS. Different test concentrations of diterpenes (100 µl) were then aseptically added to each of the microtitre plate containing cell line, where untreated cells were taken as a positive control. The plates were then incubated at 37°C with 5% CO_2_ atmosphere for 72h. The plates were checked every 24h for any signs of contamination. After 72h, the test dilutions were flicked off from each plate and 50 µl of the MTT solution (5mg/ml in PBS) was added to each well. All the microtitre plates were gently shaken and incubated at 37°C in a CO_2_ incubator for 3h. The solution was then carefully removed and 100 µl DMSO was added to each well so as to solubilize the formazan crystals. The absorbance was measured using a microplate reader at 540 nm. The percent growth inhibition was calculated using formula:

Mean OD of each test concentration

Mean OD of the positive control

% growth inhibition = 100 – x100

The concentration of the test compound (diterpenes) needed to inhibit the cell growth by 50% (IC_50_) was calculated from the dose-response curve generated for each cell line.
