# Supplementary material for: Scientific validation of the antimicrobial and antiproliferative potential of Berberis aristata DC root bark, its phytoconstituents and their biosafety
Source: AMB Express. 2019 Sep 11;9:143. doi: 10.1186/s13568-019-0868-4 (PMC6738363; doi:10.1186/s13568-019-0868-4)
Supplement: Supplementary file 3 — Additional file 3. Acute Oral Toxicity study of Berberis aristata diterpenes in Swiss albino mice-target animals, toxicity assay procedure (biochemical parameters, organ and body weight analysis, histopathological analysis). [file 13568_2019_868_MOESM3_ESM.docx]

**Scientific validation of the antimicrobial potential of *Berberis aristata* DC root bark, its phytoconstituents and their biosafety in terms of Ames test, MTT assay and Acute Oral Toxicity study**

**Henna Sood^a^, Yashwant Kumar^b^, Vipan Kumar Gupta^c^ and Daljit Singh Arora^a^***

^a^ Microbial Technology Laboratory, Department of Microbiology, Guru Nanak Dev University, Amritsar-143005, India.

^b^ National Salmonella & Escherichia Centre and Diagnostic Reagents Laboratory, Central Research Institute, Kasauli (H.P.) – 173204, India.

^c^ Department of Veterinary Pathology, Dr. G.C. Negi College of Veterinary and Animal Sciences, CSK Himachal Pradesh Krishi Vishvavidyalaya, Palampur (H.P.) - 176062, India.

Tel. No. 91-183-2258802-09 Ext. 3506, Fax No. 91-183-2258819-20

*Corresponding author: Prof. Daljit Singh Arora, Microbial Technology Laboratory, Department of Microbiology, Guru Nanak Dev University, Amritsar-143005, India

**Acute Oral Toxicity study of *Berberis aristata* diterpenes in Swiss albino mice**

**Target animals**

Healthy Swiss albino mice (males and females) weighing between 25 g to 35 g and aged 8 to 10 weeks were obtained from the animal house, Central Research Institute. The mice were divided into 4 groups: 2 test groups (6 male; 6 female) and 2 control groups (6 male; 6 female). The experimental procedure was approved by the Committee for the Purpose of Control and Supervision of Experiments on Animals (CPCSEA) (No. CPCSEA/IAEC/CRI/14-114-2016) and performed according to the Organization of Economic Co- operation and Development (OECD) guideline 420 for testing of chemicals

**Acute Toxicity Assay**

The mice were acclimatized to the animal house facility for a week prior to experimentation by housing in cages at room temperature (approx 23°C) and maintaining a 12h light/dark cycle. Drinking water and food were provided *ad libitum* throughout the experiment. Prior to dosage, the mice were fasted overnight but were allowed free access to water. Following the fasting period, body weight of the mice was determined and a single dose (5000mg/kg) was calculated in reference to the body weight, as the volume of the extracts solution given to the mice is 10 ml/kg. The experimental animals were divided into 4 groups as mentioned above and the calculated dosage was administered by oral route. Food was provided to the mice approximately an hour after the treatment. The mice were maintained under continuous observation for any signs of toxicity and mortality at 4h and 24h, and daily for a period of 14 days. During this period the surviving animals were weighed and visually observed for mortality, changes in behavioral pattern and physical appearance, injury and any signs of illness. After overnight fasting, on the 15^th^ day, the final weight of each mice was noted and were anaesthetized using xylaxine and ketamine (5mg/kg and 2.5mg/kg b.wt. respectively).

**Biochemical analysis**

The blood samples were collected via cardiac puncture in non-heparinized tubes and left at room temperature for 30min to allow clotting. The serum samples, thus collected, were analyzed for determination of alanine aminotransferase (ALT), alkaline phosphatase (ALP), aspartate aminotransferase (AST), total bilirubin (TBIL), urea and creatinine levels.

**Organ and body weight analysis**

Following blood collection, all the animals were sacrificed by overdose of anesthesia. The vital organs mainly liver, kidney and heart were removed, cleaned with saline and examined for any lesions. The individual organs were weighed and preserved in 10% buffered formalin for histopathological observations. The Organ-to-body weight index (%) was calculated as (weight of organ/ weight of the mice on the day of sacrifice) x 100.

**Histopathological analysis**

The tissue blocks (4-5mm) from each organ were placed in tissue capsule for overnight washing under slow running tap water. The blocks were then dehydrated in ascending grades of alcohol (50%, 70%, 90%, absolute alcohol) for 1hr each, and then were cleared using benzene followed by three changes of molten paraffin wax maintained at 58-60°C for tissue impregnation. Thereafter, tissues were embedded in paraffin blocks using L-moulds. After hardening of the paraffin blocks, tissue section of 4-5µm thickness were cut using a Microm microtome and were collected in tissue floatation bath containing 5% gelatin solution. The tissues were heat fixed by keeping the slides onto a hot plate for 10-15 min. The sections were stained using Haematoxylin and Eosin (H&E) (Himedia) staining and examined under light microscope for any tissue alterations due to toxicity.
